# Hyaluronic Acid—Extraction Methods, Sources and Applications

**DOI:** 10.3390/polym15163473

**Published:** 2023-08-19

**Authors:** Callejas-Quijada Graciela, Escobar-Chávez José Juan, Campos-Lozada Gieraldin, Pérez-Marroquín Xóchitl Alejandra, Aguirre-Álvarez Gabriel

**Affiliations:** 1 Instituto de Ciencias Agropecuarias, Universidad Autónoma del Estado de Hidalgo, Av. Universidad Km. 1 Rancho Universitario, Tulancingo C.P. 43600, Hidalgo, Mexico; ca290659@uaeh.edu.mx (C.-Q.G.); ca409778@uaeh.edu.mx (C.-L.G.); pe409780@uaeh.edu.mx (P.-M.X.A.); 2 Unidad de Investigación Multidisciplinaria, Laboratorio 12: Sistemas Transdérmicos, Facultad de Estudios Superiores Cuautitlán, Universidad Nacional Autónoma de México, Cuautitlán Izcalli C.P. 54714, Estado de México, Mexico; josejuanescobar@comunidad.unam.mx; 3 Uni-Collagen S.A. de C.V., Arnulfo González No. 203, El Paraíso, Tulancingo C.P. 43684, Hidalgo, Mexico

**Keywords:** hyaluronic acid (HA), glycosaminoglycans (GAG), emerging technologies (ET), molecular weight (Mw), hyaluronidases (HAS)

## Abstract

In this review, a compilation of articles in databases on the extraction methods and applications of hyaluronic acid (HA) was carried out. HA is a highly hydrated component of different tissues, including connective, epithelial, and neural. It is an anionic, linear glycosaminoglycan (GAG) primarily found in the native extracellular matrix (ECM) of soft connective tissues. Included in the review were studies on the extraction methods (chemical, enzymatical, combined) of HA, describing advantages and disadvantages as well as news methods of extraction. The applications of HA in food are addressed, including oral supplementation, biomaterials, medical research, and pharmaceutical and cosmetic industry applications. Subsequently, we included a section related to the structure and penetration routes of the skin, with emphasis on the benefits of systems for transdermal drug delivery nanocarriers as promoters of percutaneous absorption. Finally, the future trends on the applications of HA were included. This final section contains the effects before, during, and after the application of HA-based products.

## 1. Introduction

A native extracellular matrix (ECM) is constituted for proteins, including glycosaminoglycans (GAG). GAG are polysaccharides that bind covalently to a protein backbone to form proteoglycans [1].

Hyaluronic acid (HA) is a glycosaminoglycan that is found in the ECM of soft connective tissues. It is a highly hydrated component of connective, epithelial, and neural tissues, and it is present on the ECM. Unlike other GAG, HA is non-sulfated biomaterial with no capacity to bind a core protein in the formation of proteoglycans. It was discovered in 1934 and the term “hyaluronic acid” was proposed by Meyer and Palmer, who extracted this biopolymer from the body of a bovine’s eye. They named it hialoide (meaning vitreous) and uronic acid [2,3].

HA has great biocompatibility and a high affinity for water. These properties make the use of HA in various fields possible [4]. It is commonly used in protective and physiological processes, including in the healing of wounds and burns [5], in tissue regeneration, for cell differentiation, morphogenesis, angiogenesis, and inflammation.

The average molecular weight (Mw) can influence the physico-chemical properties of HA. It is an attractive building block for several applications. Some healing systems containing HA are commonly used in surgery, pharmacology, ophthalmology, dermatology, and cosmetology. In addition, this polysaccharide has been used in various nutritional supplements and cosmetics [6,7].

This review includes the description and generalities of HA, followed by methods of extraction and its applications within the food, oral supplementation, cosmetic, biomaterial, and pharmaceutical industry. It includes dermic fillers and a section dedicated to the structure and penetration routes on the skin.

## 2. Sources of Hyaluronic Acid and Methods of Extraction

### 2.1. Structure and Production of Hyaluronic Acid

**Figure 1:**
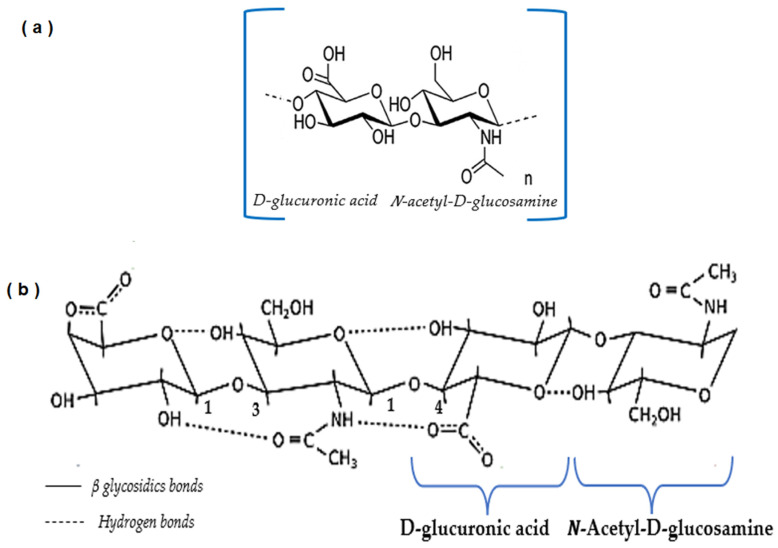
Chemical structure of HA and their components. (**a**) Structures of D-glucuronic acid and N-acetyl-D-glucosamine. (**b**) Chemical structure of the repeating disaccharide unit of HA linked together by alternating β-(1→3) and β-(1→4) glycosidic bonds. Adapted from [3,8,10,11].

Hyaluronic acid is a polysaccharide formed for disaccharide units constituted of N-acetylglucosamine and D-glucuronic acid (Figure 1a). It is composed by functional groups in HA such as carboxyl, hydroxyl, and acetyl groups enabling chemical modifications that can alter some properties. Figure 1b shows the spatial structure (two-fold helix) of HA; it is amphiphilic, with a hydrophobic patch formed by 8 CH groups. The abundant hydrophilic domains result from COOH and OH groups [8]. The disaccharide units are constituted by molar mass of 400 Da. However, the entire polymer molecule can reach up to 10,000 KDa [9].

The bioactivity of HA is highly dependent on the polymer molecular weight. Obtaining HA with a high molecular weight (Mw) is complicated due to degradation of HA during extraction, purification, storage, and sterilization processes. When HA is exposed to extreme acidic conditions, there is a disruption of the hydrogen bonds, and this can lead to random polymer degradation as well as reduction of viscosity. Production of HA with residual hyaluronidase is vulnerable to enzymatic degradation. The chemical, thermal, and enzymatic factors generate lower Mw products with wide polydispersity [12,13,14].

### 2.2. Rheological Properties

The remarkable viscoelastic and water holding properties of HA confer its unique functionality. Its high water absorption rate provides high viscosity to HA solutions at lower concentrations. The viscosity and elasticity of the hydrated polymer can vary with shear rate. A high shear rate leads to a lower viscosity and a higher elasticity. This is, in other words, the ability to store energy and facilitate recovery from deformation. The viscoelastic behavior depends on the solution conditions. The pH could lead to important changes in the viscosity of the solution based on the state of entanglement, bonding, and electrostatics [3,15]. Solutions of HA with high MW are very viscous and they show non-Newtonian flow behavior [16]. Hyaluronic acid is semi-flexible and the hydrodynamic volume of chains is fairly large. This volume in the chains is represented by water that is not bound with the polymer. Viscoelastic solutions closely mimic the synovial fluid found in joints. This is the reason why most of the natural viscoelastic properties of synovial fluid can be attributed to the concentration of hyaluronic acid contained in this fluid [6].

### 2.3. Commercial Production Systems of HA

#### 2.3.1. Production of HA from Animals

Since HA was discovered in 1930, the extraction of HA from animal tissues has been developed in order to identify, characterize, and elucidate its biological potential. It is present in almost all tissues of vertebrates, including the vitreous body of the eye, synovial fluid, pig skin, the pericardial fluid of the rabbit, the cartilage of sharks, and other marine animals [17].

#### 2.3.2. Production of HA from Microorganisms

Hyaluronic acid is part of a component of the extracellular capsule formed by some microorganisms, such as *Streptococcus*, that serves not only for adherence and protection but also as a molecular imitation used to evade the host immune system during its infection process [18]. Commercial production was performed with *Streptococcus equi* and *Streptococcus zooepidemicus*. The *S. equi* produced HA with a lower molecular weight than *S. zooepidemicus* [19,20,21,22,23,24].

The first isolation of hyaluronic acid from hemolytic streptococci was carried out in 1990. The enzymes from *Streptococcus* species extend the HA chain, including human hyaluronidases (HAS) enzymes [3,10]. The main advantage of Streptococci strains for hyaluronan production is the use glucose as a carbon source. Some studies reported that the yield of hyaluronan on glucose under aerobic fermentation conditions is between 5–10%. This value is higher than yields for complex polysaccharides in lactic acid bacteria. The use of carbon sources such as starch and lactose are available at low costs [10].

Microbial HA production is also known as “vegan hyaluronic acid” or “hyaluronic acid from plant origin”. *Streptococcus equi* subsp. *zooepidemicus* has been used for degradation of residues from green coffee and tequila agave and others agricultural resource derivatives. The obtained extracts are applied to new and commercial products as Greenluronic^®^ for improvement of joint protections [25,26].

#### 2.3.3. Cell-Free Methods of HA Production

HA has been produced at the research-scale in cell-free in vitro systems that utilize purified hyaluronidases enzymes (HAS). These need to be tightly bound to the cell membrane. *Pasteurella multocida* can synthesize HA in a cell-free system and produces HAS that does not require membrane association. The scientific community is working hard on the development of membrane-bound HAS for cell-free production [27,28] because the current cell-free systems do not have the capacity to produce at large scales [3].

### 2.4. Methods of Extraction of Hyaluronic Acid

Characterization of HA has been carried out in vitro and in vivo HA synthesis from bacterial strains or cell free. Unfortunately, there are some limitations, such as short HA chains as well as wide polydispersity in the Mw of the final product. This problem remains an issue as the distribution is heavily controlled by reaction stoichiometry and other culture conditions [29].

The isolation of HA involves protocols that are similar to those historically employed in the purification of DNA. It depends on the nature of the sample; in solid tissues, hyaluronic acid is extracted into soluble form. After that, HA must be liberated from proteins. This step can be accomplished by digestion with a protease or by denaturation of the protein with chloroform. Lipids are removed using organic solvent mixtures. Finally, to remove the low molecular mass contaminants, it may require dialysis or precipitation of the HA with ethanol or isopropyl alcohol [14].

The extraction of HA from solid tissue includes several steps: digestion (protease), boiling (denature enzyme), centrifugation, extraction with chloroform, centrifugation, dialysis, precipitation with ethanol, centrifugation, redissolution, digestion (such as Benzonase), and boiling for enzyme denaturation.

This purification cannot remove other glycosaminoglycans and it could be necessary to apply the anion exchange chromatography process [30,31].

There are techniques oriented to extract HA by using detergents, enzymes, and/or solvents to breakdown the structure and isolate polysaccharide complexes present in tissues. These techniques are based on the chemical hydrolysis of the tissue to ensure the disruption of the proteoglycan core after the removal of proteins and recovery of the polymer [32,33].

The isolation of compounds from marine and terrestrial sources includes enzyme hydrolysis for the recovery of different active materials such as proteins and polysaccharides [32].

Table 1 shows different processes of extraction and purification methods developed for the isolation of HA. Two or more methods were applied to extract HA from marine and terrestrial animal by-products to ensure the maximum exploitation of wastes. Some examples include shark, stingray (liver), swordfish, and tuna [34,35,36].

HA can be extracted from different parts of the organisms (cartilage, head, eyes, fins, and skin). Terrestrial by-products (waste tissues) are generated daily from slaughterhouses or other food industries. Some studies have indicated that the average animal waste per ton of total weight of killed animals is near 2.3 kg for pig waste and 275 kg in bovine source. This accounts for 4% and 27.5% of the animal weight, respectively [37,38].

Rooster comb, vitreous humor, umbilical cord, and synovial fluid are useful raw materials for HA extraction. However, the highest concentrations of HA have been reported as follows: cattle, pig, and sheep synovial fluid (up to 40 g/L) [39], rooster comb (39.8 g/kg), and wattle tissue (17.9 g/kg) [40].

Da Rosa et al. [41] conducted an experimental work that consisted of the extraction of HA from chicken crest. This extraction consisted of a proteolytic digestion with papain; the content hexuronic acid was determined by using the carbazole method. The results showed that this extraction method was effective as there was a large molecular mass of HA.

Kulkarni et al. [42] worked on the extraction of HA from chicken combs. Several qualitative tests (UV absorption, endotoxin detection assay, protein contamination of polymer by SDS-PAGE) were included in the study. Results showed that the final extract was not contaminated with the protein, resulting in pure hyaluronic acid.

Kang et al. [43] reported that 500 g of frozen rooster combs yielded about 500 mg of dried HA. The molecular size was characterized using an asymmetric flow field-flow fractionation (AsFlFFF) coupled with multiangle light scattering (MALLS) and refractive index (RI) detector (AsFlFFF-MALS-RI). It was difficult to analyze HA by the conventional three-step operation of AsFlFFF due to the adsorption of HA onto the membrane. The online combination of AsFlFFF with MALS provided useful information on molecular weight distribution. A simplified operation of AsFlFFF-MALS-RI seems to provide a useful tool for analysis of highly viscous and high Mw macromolecules such as HA.

Traditional techniques for the isolation of glycosaminoglycans suggest the use of enzymes (papain, trypsin, pepsin, and pronase) for the degradation of the tissue and the breakdown of the protein fractions [32]. When HA is extracted from rooster/chicken combs or mollusk bivalve, tissues are degreased using acetone; then, papain, used as an enzyme, is followed by boiling to denature the enzyme. The sample obtained in this manner is precipitated with ethanol and sodium acetate (NaOAc) [36,40,44,45,46].

Űrgeová and Vulganová [47] realized the extraction of hyaluronic acid from the membranes of eggshell using various enzymes. For isolation, they employed different enzymes, including pepsin, trypsin, and papain. The best yield was reported in trypsin treatment with 44.82 mg/g dry eggshell membranes.

Organic solvents and inorganic salts, such as NaOAc and CPC (Cetylpyridinium chloride), have been employed to extract HA from bovine synovial fluid by the formation of HA-CPC complex [42,43,48,49,50,51]. Amagai et al. [34] extracted hyaluronic acid from the eyeball of bigeye tuna in cold conditions. Cetylpyridinium chloride was applied to separate mucopolysaccharide. The polymer obtained was characterized by gel permeation chromatography and viscometry. The results reported lower values when compared to other sources.

Balazs [52] also isolated this polymer from the vitreous humor of owl monkey eyes. The treatment with solvent (chloroform) produced a two-phase mixture for analysis of the liquid-liquid extraction.

Khanmohammadi et al. [53] worked in the extraction of hyaluronic acid from eggshell by using acetic acid solution 8 M. Samples were precipitated by centrifugation and purified with isopropanol and then suspended in 1 L of 3% sodium acetate. The purification step was then followed with silica gel at 2% final concentration. Activated charcoal was included for separation. Filtration was applied with membranes of 0.45 mm and 0.2 mm, followed by the lyophilization process in order to obtain a purified product.

Some purification procedures, such as ultrafiltration or diafiltration, are size-based methods used to remove the impurities from and concentrate the polymer in solution. Additional techniques include ion exchange and dialysis, which have been employed for protein separation and purification as a final step for the purification [34].

Organic solvents such as chloroform and methanol are commonly used for the separation of proteins and lipids after the application of papain in the extraction of HA from chicken combs. Chloroform was also used as a solvent in the extraction of HA from rooster combs without enzymes [42,45]. Abdallah et al. [32] used the application of ultrafiltration-diafiltration to ensure a high purity of HA.

Emergent technologies have been applied to the final step in order to eliminate the use of these solvents (ethanol, chloroform, sodium acetate solution) or ion exchange separation [32]. The use of emerging technologies (ET) such as ultrasound and microwave offer an option to extract HA, thereby reducing the environmental impact and economic issues and obtaining high purity HA [54,55]. Ultrasound is a process that uses the energy of sound waves that are generated at a frequency above the threshold of human hearing. The effects of ultrasound in a liquid system are due to the phenomenon known as cavitation [56]. Aguirre-Alvarez [57] patented a collagen extraction process using high-intensity ultrasound. The extraction assisted with ultrasound reported higher amounts of collagen and a higher purity of this biomaterial when compared to conventional extractions with acid and alkali products.

Chemat [58] reported significant benefits on the use of ultrasound technique versus conventional heating methods for the HA extraction in terms of low energy consumption, shortened treatment time, less solvent usage, increased safety of the operators, increased yield, and non-thermal technology.

Microwave irradiation is a technique that also shorten the extraction times and reduce organic solvent consumption. Hafsa et al. [59] realized the chemical extraction of HA from rooster combs assisted with ultrasonic degradation in order to obtain low molecular weight HA with important antioxidant and antiglycation activities.

**Table 1:** Sources and Methods of extraction of Hyaluronic acid (HA).

Source	Method	Conditions Used	Concentration	Reference
Tuna (Eyeballs)	Chemical extraction	-Treated: 3% CPC/15 min at 4 °C. -Precipitation: 0.4 M NaCl to dissociate the HA-CPC. -Centrifuged: 2.22 × 10^3^ g/15 min at 4 °C. Resuspended: 0.1 M Tris–HCl (pH 7.7) with mycolysin 24 h/37 °C. -Dialysis (for 2 days, distiller water)	0.42 g/L vitreous humor	[34]
Swordfish (Vitreous humor)	Chemical extraction	Alkaline process: NaOH concentration 0.45, 0.85 M. Ultrafiltration-diafiltration: plate polysulfone membranes cut-off at 35 °C. Protein electrodeposition: 2 platinum electrodes of 50 cm length and prepared in spiral/cylindric at 10–40 mA.	0.055 g/L HA	[35]
Shark (vitreous humor)	Chemical extraction	-Alkaline process: NaOH concentration 0.45, 0.85 M. -Ultrafiltration-diafiltration: plate polysulfone membranes cut-off at 35 °C. -Protein electrodeposition: 2 platinum electrodes of 50 cm length and prepared in spiral/cylindric at 10–40 mA.	0.3 g/L HA	[35]
Stingray (Liver)	Chemical- Enzymatic extraction	-Defatted: acetone and dried at 60 °C/24 h. -Pellet in 100 mM NaOAc buffer pH 5.5 containing 5 mM EDTA, 5 mM cysteine. -Digestion: papain, incubated for 24 h at 60 °C in a stirrer. Precipitation: centrifuged 5000× *g* for 15 min and 3 volumes of ethanol saturated with NaOAc. Dried: at 60 °C for 6 h.	6.1 mg HA/g dry weight of tissue	[36]
Pig (Sinovial fluid) Sheep (Sinovial fluid)	Chemical Enzymatic extraction	-Extraction chloroform and NaCl -Digestion: Trypsin-Pronase chloroform treatment and filtration at 37 °C.	Less 5 µg of protein per milligram of HA	[39]
Wattle	Enzymatic extraction	Papain Dialysis and cellulose acetate electrophoresis	17.9 μg/ mg	[40,52]
Rooster comb	Chemical Enzymatic extraction	-Defatted: Acetone and dried at 80 °C. -Digestion: twice-crystallized papain in 1 mL of 0.1 M sodium phosphate buffer containing 0.005 M EDTA, 0.005 M cysteine hydrochloride, 0.02% sodium azide having a pH of 6.5. 65 °C for 4 h. -Dialysis: dialysis tubing (molecular mass cutoff, 6000–8000 Da) for 24 h.	39.8 μg/mg	[40]
Chicken comb 50:50 male and female	Chemical Enzymatic Extraction	Dehydration: acetone. Extraction/delipidation (chloroform and methanol solution (2:1 *v*/*v*) for 24 h at 25 °C). Extraction: Papain digestion buffer (20 mg/mL), ethanol to purification and centrifugation.	Dry material 15 g hexuronic acid/mg dry tissue	[41,45,51]
Rooster comb	Chemical extraction	-Defatted acetone (3 intervals) each 24 h at 8 °C. -Extraction: NaOAc 5% -Precipitation: sodium saline citrate.	*	[42]
Rooster comb	Chemical extraction	-Defatted: acetone -Extraction: NaOAc 5% -Precipitation: chloroform–amyl alcohol -Dialysis	1 mg/g of frozen rooster comb	[43]
Mollusk- Bivalve	Enzymatic extraction	-Defatted with acetone. -Centrifugation and pellets dried. -Digestion: Buffer (100 mM NaOAc pH 5.5, 5 mM EDTA and cysteine), papain (100 mg/g of tissue). -Samples: 10 mL of 0.05 M NaCl and centrifugation. -Anion exchange: column chromatography (DEAE cellulose).	4.2 mg/g dry weight of tissue	[46]
Eggshell- Membrane	Enzymatic extraction	-Hydrolysis: Pepsin, trypsin, and papain at 37 °C/5 h. pH 3	Papain: 39.02 mg/g Trypsin: 44.83 mg/g Pepsin: 29.70 mg/g	[47]
Bovine Synovial fluid	Chemical extraction	-Diluted in water and dissolved in CPC. -Precipitation: NaCl and ethanol 40% *v*/*v* Fuller’s earth (50 g of original material in 300 mL of phosphate buffer). -Dialysis: distiller water, 12 h at 4 °C.	250 mg/L	[48,53]
Eggshell- membrane	Enzymatic extraction	Treated: yeast enzyme complex pH to 7.2; CPC at 1:60 (*v*/*v*); centrifugation; ethanol to filtered HA solution 2:1 ratio, centrifugated; dissolved in 0.2 M NaCl in 0.2 M phosphate buffer, pH 7.2; ethanol precipitation, filtration, and acetone wash.	* Hyaluronan dry powder	[49]
Rooster combs	Enzymatic extraction	Water 100 °C. Papain; ultrafiltration in 40% water-ethanol mixture.	* Lyophilized powder	[50]
Rooster combs	Chemical extraction	Water extract heating at 90–100 °C; lipid removal; filtration; treatment with activated carbon.	* Lyophilized powder	[50]
Rooster combs	Chemical extraction	Physiological solution, 80–90 °C, 2 extractions. Filtration: precipitation acetic acid with NaOH to pH 7–7.3, heating to 80–90 °C; repeatable filtration.	* Lyophilized powder free from nucleic acids.	[50]
Rooster combs	Chemical extraction	3 extractions: water Precipitation: trichloroacetic acid from the extract volume at 20–22 °C form 1–2 h; lipid and water removal with acetone and ether three times.	* Lyophilized powder	[50]
Rooster combs	Chemical extraction	1–15% solution of NaCl at 60 °C, 18 h. Yield 1.92% from the stating material, centrifugation; lyophilization.	Fibre-like white substance; protein content 9–24%	[50]
Rooster combs	Ultrasound-Chemical extraction	Treated: ethanol and ultrasound (16–20 kHz 20–25 min). Extraction conditions: water at 45–50 °C, 20–25 min 55% of HA. Vacuum filtration: HA 95% precipitation with ethanol at the ratio 1:3, drying.	* Hyaluronan dry powder	[50]
Rooster combs- umbilical cord	Chemical extraction	Grinded raw material frozen to (−20–70 °C), 2 parts of water by weight added and mixture heated 15–25 min at 95–100 °C.	* Hyaluronan dry powder	[50]
Rooster combs	Chemical extraction	Treated collagenase 0.03–0.04% to the tissue weight for 45–50 min, 45–50 °C, pH 6.8–7. Precipitation: ethanol at the ratio 1:3; vacuum filtration, vacuum drying or sublimation.	* Hyaluronan dry powder of solution	[50]
Rooster combs	Enzymatic extraction	Frozen tissue treated with water 55 °C. Proteolysis: 3.5 h at 37 °C. Filtration (5.6 g/1 kg of the tissue). Precipitation: dissolved 30% ethanol with NaCl, reprecipitated (ethanol).	* Hyaluronan dry powder	[50]
Rooster combs	Enzymatic extraction	Combs boiled: 4 h at 50 °C and pH 7.5 with Pronase. Yield: 6.7 g/1 kg tissue. Filtration: CPC. Precipitant: 30% ethanol and NaCl.	* Hyaluronan dry powder	[50]
Rooster-chicken combs	Chemical extraction	Water pH 3–4, 90–100 °C, 50 min. Treatment with activated carbon then cellulose; filtration.	* Lyophilized	[50]
Rooster-chicken combs	Chemical extraction	Extractions: water. Treatment chloroform. Precipitation: ethanol.	* Lyophilized	[50]
Rooster /chicken combs	Chemical extraction	Wash (ethanol, chloroform). Extraction: 3.5 volumes of water, acidified (pH 3–4 at 90–100 °C, 40–60 min), yield 0.09%. Extracts (filtered), proteins (60–80 °C). Filtration: 40 °C through polyvinylchloride membranes.	* Powder dried	[50]
Chicken combs	Chemical extraction	Solution of tertbutyl alcohol twice (5–25%). NaCl to creation of two-phase system precipitation (ethanol).	* White amorphous powder	[50]
Owl monkey (Eyes)	Chemical extraction	Use of organic sodium salt Dialysis	291.8 μg/mL vitreous humor	[51]
Chicken comb (Eyes)	Chemical Extraction	Sodium salt, Dialysis	469.9 μg/ mL vitreous humor	[51]
Rooster combs	Chemical extraction	Extraction: water. Treatments with mixture chloroform and NaCl 5 °C, 3–5 h; treatment: Pronase. Precipitation: ethanol.	* Lyophilized powder	[52]
Owl monkey (Eyes)	Chemical extraction	-Use of organic solvents Salts: NaCl 1 M, CPC and ethanol. -Deproteinized: chloroform treatment.	3.97 g	[52]
Eggshell- membrane	Chemical extraction	-Extraction: Acetic acid 4 M and isopropanol. -Precipitation: centrifugation at 18,000 rpm, 20 min at 4 °C Washed: NaOAc 3%	5.3 mg HA/g Eggshell	[53]
Wattle	Enzymatic extraction	Pronase chloroform treatment and ion exchange chromatography	Yield > 90% with respect to hexuronic acid	[60]
Rooster comb	Chemical extraction	Organic solvent and NaOAc, chloroform treatment	*	[61]

HA (Hyaluronic acid), NaOAc (Sodium acetate), CPC (Cetylpyridinium Chloride) NaCl (Sodium Chloride), Sodium Hydroxide (NaOH), EDTA (Ethylenediamine tetra acetate), Yield non specified by the authors (*).

## 3. Applications

### 3.1. Food Industry

Determination of hyaluronic acid in supplements is complicated due to the low polymer content and the presence of several other components such as proteins (collagen), vitamins, and plant extracts [62]. As illustrated in Table 2, Zając et al. [63] studied HA as a food additive for meat emulsions. They reported that the addition of 0.05–0.1 g of HA reduced the yield and stability of meat emulsion after 14 days of storage under vacuum conditions. HA caused water outflow from the product and decreased the sensory evaluation of sausages.

HA has been added to some dairy products, including yoghurt. A three month nutritional study confirmed that oral administration of HA in healthy individuals with joint discomfort of the knee provided an enhancement in muscle strength [64]. Sutariya et al. [65] studied the effect of HA in milk at several concentrations. The physicochemical properties of milk showed no effect on the viscosity.

### 3.2. Oral Supplementation

Oral HA supplementation has become popular as an excellent anti-aging product; this is due to its provision of nutrients to the skin. HA improves skin physiology and appearance, resulting in better hydration, elasticity, wrinkle reduction, and skin rejuvenation [66]. In addition to oral supplementation, there is a category classified as “nutricosmetics” that can be described “as the consumption of food or oral supplements to produce an appearance benefit”. These are also called “beauty pills”, “beauty from within”, and even “oral cosmetics”. Nutricosmetics is defined as the combination of nutrition, health, and cosmetics through the consumption of functional foods and nutraceuticals. The “beauty from within” can be provided by oral supplements, “beauty foods”, or “anti-aging cocktails” [67].

Table 3 shows the applications of HA as a functional supplementation food with positive effects on the skin. Hsu et al. [68] carried out a randomized test based on the daily intake (120 mg/capsule) of HA for 12 weeks in 40 healthy individuals. Their evaluated conditions (stratum corneum water content, transepidermal water loss, and elasticity) were improved in the HA group when compared with the control group. The evaluation indicated that oral ingestion of HA could suppress some wrinkles and improve skin condition. Oe et al. [69] evaluated anti-wrinkle and Mw effects in oral ingestion of HA at different Mw (2 kDa, 300 kDa). In the study, 120 mg/day of HA were ingested for 12 weeks by Japanese individuals aged 22–59 years. The study showed that after 8 weeks of treatment, oral ingestion of 300 kDa HA inhibited wrinkles and improved skin condition when compared to the placebo group. After 12 weeks, both treatments reported that skin luster and suppleness were significantly improved.

Zhao et al. [70] caried out a study in vitro to simulate the fermentation of HA in the colon. The objective was to investigate the interaction between HA with several molecular weights and the intestinal microbiota. They performed in vitro fermentation of human-derived feces for three samples of HA (32.3 kDa, 411 kDa, and 1510 kDa). The findings were that gut microbiota can utilize the different concentrations and produce large amounts of short-chain fatty acids. The three samples promoted the growth of *Bacteroides*, *Parabacteroides*, and *Faecalibacterium*. However, the lowest Mw sample (32.3 KDa) significantly promoted the growth of *Bacteroides*. The results of this study show the scientific basis for the targeted regulation of gut microbiota by oral HA ingestion.

Manfredi et al. [71] evaluated the efficacy of oral preparations of hyaluronic acid, chondroitin sulfate, curcumin, and quercetin (Ialuril^®^ Soft Gels) used to decrease the degree the severity of low urinary tract symptoms. All subjects enrolled in the experiments had a history of non-muscle-invasive bladder cancer after intravesical chemotherapy. Patients were evaluated at baseline and after several months (1, 4, 7, 13) of intravesical chemotherapy. They were randomized into two groups (intervention vs. control) and all subjects underwent oral administration. The international prostate symptom score and the 0–100 visual analogue scale were used to assess the efficacy of the treatment. The median score was significantly lower in the intervention group when compared to the control group. The results showed this formulation could be an effective and safe therapy against chemical cystitis in patients receiving intravesical chemotherapy for bladder cancer.

Sifre et al. [72] tested a basic formula (chondroitin sulfate, glucosamine hydrochloride) and native type II collagen in 54 female New Zealand rabbits. They were classified into three groups: control, basic formula, and basic formula-native type II collagen. Each group was subdivided into three subgroups. Rabbits developed degenerative changes associated with osteoarthritis and the formulation basic formula-native type II collagen improved values on macroscopic evaluation when compared to the control and only basic formula groups. Results showed that oral administration of the evaluated materials was safe, and that the addition of native type II collagen increased its efficacy.

When HA is taken orally, it breaks down in the stomach and is rendered useless for the skin. However, glucosamine supplements (HA, dermatan sulfate, heparin, keratin sulfate) can help to increase the production of HA in the skin [73]. Glycosaminoglycans, such as chondroitin sulfate, are easily degraded into oligosaccharides by bacteria in the large intestine and are eventually absorbed [74]. However, absorption of HA is unclear due to its distribution throughout the entire body. Under this argument, Kimura et al. [75] evaluated the excretion into the feces, degradation in the intestinal tract, absorption through the large intestine, and translocation to the blood and skin in a mice model. They suggested that consumption of HA leads to degradation into oligosaccharides by intestinal bacteria. Oligosaccharide HA is absorbed in the large intestine and is distributed in the body. This information could be useful in the study of various dietary supplements as well as medicinal and cosmetic preparations containing hyaluronic acid. This active substance promotes the regeneration of cartilage tissue, skin softening, and wound healing [7].

Fritz et al. [76] investigated the mechanisms of action as well as the physiological role in dietary supplements with L-arginine, HA, collagen, and vitamin C against musculoskeletal complaints. The effectiveness of the supplement in elderly patients was evaluated. This study involved 30 people divided into two groups (study and control); both groups consumed 25 mL of the supplement per day. This dose contained 500 mg vitamin C, 10,000 mg collagen, 40 mg HA, 1075 mg L-arginine, and 1074 mg glycine. The results demonstrated that the blood lipid levels changed; low-density lipoprotein decreased, high-density lipoprotein increased, and the ratio significantly decreased in both groups. For the experimental group, there were significant changes attributed to the addition of regular physical activity. Knee and hip joint range of motion showed significant improvements. The investigated dietary supplement can be used for joint, cardiovascular, and nutritional health promotion as well as for prophylactic purposes.

Mirzayeva et al. [62] identified the inclusion of hyaluronic acid in three food supplements (powder, tablets, and capsules) produced using three different techniques: gravimetrically, spectrophotometrically, and isotachophoresis. They reported that the combination of the appropriate preparative along the analytical steps led to the effective quantification and evaluation of HA composition. The development of these procedures could be used to analyze other products containing hyaluronic acid for the regeneration of tissue and wound healing.

**Table 3:** Hyaluronic acid (HA) applications as functional supplementation food with positive effect on the skin.

Product	Source	Functionality	Reference
Capsules	HA (Habest^®^) 95% Purity	Trial HA (120 mg) intake for 12 weeks in 40 healthy individuals Asian that consume oral ingestion	[68]
Capsules	HA (Hyabest^®^) 95% Purity	Effect of oral intake of HA for 12 weeks with individuals Japanese.	[69]
Oral preparation	HA, CS, curcumin, and quercetin	Therapy against cystitis in patients receiving intravesical chemotherapy for bladder cancer.	[71]
Oral administration	CS, GlHCl, HA, native collagen type II	Beneficial joint health effects of basic formula (CS + GlHCl + HA) and basic formula plus native collagen type II which results in even greater efficacy.	[72]
Dry powder	HA of two Mw (Kewpie Corporation, Tokyo, Japan)	Degradation and absorption of HA in excretion into the feces, intestinal tract, large intestine, and translocation to the blood and skin were examined.	[75]

Chondroitin sulfate (CS), HA (Hyaluronic acid), Glucosamine Hydrochloride (GlHCl).

### 3.3. Cosmetic Industry

The hydration effect in cosmetics formulations indicates the amount of HA synthesized. The epidermis synthesizes four times more HA when compared to the dermis. The hygroscopic properties of the skin are relevant for hydration of the deep layers of the epidermis. HA is very helpful for hydration of the skin and it regenerates the skin when applied topically. Some commercial formulations with different concentrations of HA can be obtained in the market, and some could be used for healing treatments with the advantage of reduced skin irritation. Others barrier products containing HA have been approved by the FDA for skin problems. These problems include nasolabial folds, wrinkles, skin hydration, collagen stimulation, anti-aging, and skin augmentation. HA has also been applied to a wide range of cosmetic formulations. Its strong water-holding capability is the main reason why this biopolymer is commonly used for the maintenance of the skin in terms of turgidity, moisture, and elasticity [15,77,78,79].

HA has been used in several different forms, including creams, serums, gels, lotions, intra-dermal filler injections, and facial fillers. Its uses in these forms are related to its capability for face rejuvenation, collagen stimulation, and tissue augmentation [80].

The skin becomes drier, thinner, and looser, leading to wrinkling, when cells lose their ability to produce HA. This is because this versatile material has relevant properties linked to the retention of water molecules [81]. For this reason, HA is one of the most widely used active ingredients in cosmetic formulations (Baumann and Saghari, 2009). Adding HA to the skin promotes water into the skin and reduces wrinkles significantly [73].

The skin is the most extensive route for systemic and topical administration of drugs and bioactive components. Transdermal delivery is a less invasive approach for medication and cosmetic administration; it provides controlled drug distribution, less frequent dosing, patient compliance, and prevention of first pass metabolism.

Ethosomal systems, such as newer lipid vesicular carriers, are used because of their ability to carry medicinal substances with physicochemical qualities throughout the skin and deep skin layers. They can be placed in gels, patches, or lotions [82]. Chen et al. [83] prepared an ethosomal carrier system encapsulating HA in order to enhance penetration into the active epidermis and dermis. The main objective of this carrier system is to penetrate the dense structure of the stratum corneum. Kong et al. [84] developed an alcohol-free oil-water HA nano emulsions with skin permeation capacity. The diffusion of the emulsion and the delivery of HA were carried out through follicular and intercellular routes.

Table 4 illustrates commercial cosmetics that incorporate HA and their derivatives. Tokudome et al. [85] studied the passive delivery of HA nanoparticles (HANP) into the skin. These nanomaterials were obtained using the polyion complex method. The results showed that HANP significantly reduced transepidermal water loss. It was observed that HA was delivered in an effective way by nanoparticles contributing to barrier recovery after UV irradiation treatment.

Researchers have been concerned with improving the appearance of the skin by using patches for the improvement of facial wrinkles and overall skin condition [86]. Choi et al. [87] worked with 34 Korean female subjects to treat their crow’s feet wrinkles. A HA microneedle patch was applied to one side of the face and HA essence was applied to the other side. The patch was safer, did not cause skin irritation, and was more effective for wrinkle improvement when compared to the HA essence.

The main advantage of this system is the penetration of active agents into the skin without pain. The patch created micro conduits through the stratum corneum without disrupting nerves and blood vessels of dermis [88]. HA-based microneedles of adenosine and a mixture of bioactive proteins were developed by Avcil et al. [89]. Wrinkle depths exhibited a 26% improvement. This treatment catalyzed elastin and collagen synthesis. The efficacy of HA depends on molecular weight. This parameter produces important effects, including hydrating, regenerating, and anti-ageing [7,90,91]. Microneedles have not only been used in the treatment of face wrinkles. They have been also studied as an alternative treatment to hypertrophic scarring. For example, bleomycin is the most common treatment, but it has the disadvantage of pain and being a long-term process [92]. Jan et al. [91] developed a system using bleomycin-loaded dissolving microneedles prepared with HA. The HA matrix maintains the stability and activity of bleomycin and inhibits the proliferation of human hypertrophic scar fibroblasts. Xie et al. [93] also used Bleomycin-loaded dissolving microneedles. This treatment inhibited the proliferation of human hypertrophic scar fibroblasts as well as the secretion of transforming growth factor-β (TGF-β1) in vitro.

Cosmetics containing HA also contain different plant extracts, vitamins, amino acids, proteins, saccharides, probiotics, and even gold. Some consumers have become more interested in natural sources and sustainable ingredients due to their compatibility with hair follicles. However, sulfates continue to be used as cleansing agents due to their effectiveness and low cost [94].

**Table 4:** Commercially available cosmetics incorporating HA and their derivatives.

Product	Source	Functionality	Reference
Nanoparticles	HA Commercial	Effectively delivered by nanoparticles than passive diffusion and could contribute to barrier recovery following UV irradiation.	[85]
Microneedles	HA Commercial	Verify the face skin improvement effect and safety of a novel cosmetic microneedle patch.	[86]
Microneedles	HA Commercial	Effective than the HA essence for wrinkle improvement and safe.	[87]
Microneedles	HA Commercial	Skin rejuvenation due to its water-retaining ability and viscoelastic nature.	[89]
Microneedles	Adenosine encapsulated high and low molecular weight HA	It was analyzed the skin improvement and the patch which HMw patch showed the better effect than LMw HA patch with the similar adenosine doses.	[91]
Dissolving Microneedle array	HA Commercial/ Hydroxypropyl-β-cyclodextrin Triamcinolone acetonide	Alternative treatment to hypertrophic scar was evaluated in a model in rabbits the delivery of administration strategy.	[93,95]

Hyaluronic acid (HA), Molecular weight (Mw), Low Molecular weight (LMw), Hight Molecular weight (HMw).

### 3.4. Dermic Filler

The lips and the mouth have a functional importance because they participate in vocalization, mastication, and the aesthetics of the face. When age is advanced, the skin show effects of gravity, laugh lines, smile lines, crow’s feet, and other facial creases. The skin loses hydration due to a decreased natural production of HA. Dermal fillers containing HA are commonly used as an alternative to surgical treatment. Unfortunately, there is a lack of data to compare the range of HA products and how they relate to tissue performance [96].

Soft tissue fillers could help to fill these lines and creases temporarily. These fillers produce a smoother, more youthful appearance of the skin. The application of these dermic fillers are non-permanent, have minimal side effects, are painless upon injection, and have a low cost [82,97]. HA fillers represent about 80% of all fillers used for rejuvenation and volume correction [98].

Fundarò et al. [98] published a review of the rheological properties that can help clinicians understand filler characteristics and clinical outcomes. They identified nine characteristics in filler science. Some of them could be mentioned as follows: rheological characteristics (elastics modulus, viscous modulus, complex modulus, cohesivity, complex viscosity) and physicochemical characteristics (hydration capacity, HA concentration, degree crosslinking). Fillers for deep injection are generally defined as “harder” and fillers for fine lines as “softer”. Soft fillers are thought to have lower viscosity and elasticity and have the tendency to spread into soft tissue (i.e., ideal for fine lines and wrinkles).

A consequence of the use of HA based dermic fillers consists in non-vascular inflammatory adverse effects when patients receive injections [99]. Lee et al. [100] analyzed impurities in HA fillers. The particles were counted when degraded by hyaluronidase. Afterward, samples were identified using energy-dispersive X-ray spectroscopy, identifying impurity particles >10 µm.

Rho et al. [101] used 36 female patients older than 18 years old for their study. They used two different HA fillers with low and intermediate cross-link density (about 1–2% and 2–3%, respectively). These products contained 20 mg/mL of HA and 0.3% lidocaine. The changes of columella labial angle were measured and analyzed. There was a statistically significant increase in mean lip volume and lip projection at 4 and 12 weeks after injection. Three-dimensional photographs of face were taken before the procedure in order to follow-up the volumetric changes after lip filler injections. Lips injected with HA filler of intermediate cross-link density resulted in more acute angles than lips injected with lightly crosslinked HA. These findings imply that lip injections with HA fillers with intermediate cross-linking density can be a good option in patients who want a prominent upper lip lift.

### 3.5. Biomaterials, Pharmaceutical and Delivery Systems

The use of biomaterials can be applied to wound healing treatments. It implies the use of any substance that has been engineered to interact with a specific biological system. Hyaluronic acid-based scaffolds hold the structures that support and can promote cell and tissue ingrowth through biodegradable structures such as hydrogels. They are widely used within the medical industry for various therapeutic purposes, including bone tissue, space-filling, nerve and brain tissue repair, cell delivery, and muscle regeneration [102].

Table 5 shows the application of HA for the preparation of different biomaterials. He et al. [103] prepared HA-based cryogel scaffolds to promote regeneration of cartilage tissue. They reported that the fabricated cryogels resulted in a macroporus material with a high interconnected network. These characteristics provided an excellent conducive environment for chondrocytes stability, enhancing cell proliferation and production of cartilage extracellular matrix glycosaminoglycans.

Lima et al. [104] showed that surface chemistry and morphology are critical factors for the development of biomaterials. These materials can be applied to several cell adhesion applications, including rapid diagnostic, cell signaling, and biosensing mechanisms.

HA is employed in composite coating in the field of cardiovascular biomaterials biodegradables. Yu et al. [105] reported the preparation of a composite coating as an efficient approach against corrosion resistance in a composite. This coating can support the normal growth of endothelial cells and inhibit inflammation response.

HA has been used as a primary component in nanomaterials. An example of these applications are the nanoparticles that have wide-spread applications in various sectors. In medicine, nanoparticles are very efficient for drug delivery, screening of various diseases, and tissue engineering [106]. For treatment of diseases, sodium hyaluronate is an injectable high Mw HA used for its disease-modifying effects in mild osteoarthritis of the knee [107].

Kang et al. [108] have focused on the improvement of self-assembled hyaluronic acid nanoparticles (HA-NP) as a therapeutic agent for osteoarthritis (OA) treatment against the effect of high Mw HA. They showed that intra-articular injection of HA-NP attenuated the effects of CD44 expression and protected joint cartilage against OA.

Lierova et al. [109] evaluated nanoparticles (HA-NPs) in vitro. These nanoparticles were administered before irradiation and their effects on the acute and chronic phases of radiation-induced pulmonary injury were measured. They concluded that the application of HA-NPs diminished detrimental radiation-induced processes in lung tissue and decreased lung fibrosis.

HA is widely used in biomedical applications due to its excellent biocompatibility. Some studies have reported the preparation of five hyaluronic acid hydrogels based on the mixing of high and low molecular weight hyaluronic acid at different ratios and cross-linked with 1,4-butanediol diglycidyl ether. Treatment B, prepared with 10% HA (4:1 HMw-LMw), showed the best properties for regenerative medicine and tissue engineering [110].

Additional work based on the preparation of hydrogels is the research developed by Si et al. [111], who conducted 3D bioprinting with double crosslinking HA hydrogels. These materials were prepared with modified hyaluronic acid-methacrylic anhydride (HA-MA) and hyaluronic acid-3,3-dithiobis (propionylhydrazide) (HA-SH) and crosslinked with ultraviolet light. The authors concluded that the HA-MA hydrogel showed higher swelling ratio and faster degradation rate. However, the HA-SH demonstrated higher biocompatibility.

Hu et al. [112] worked on 3D bioprinting of sulfate chondroitin, gelatin, HA, and graphene hybrid osteochondral scaffolds. This study included the preparation bio-inks with different proportions of three biomaterials: gelatin, chitosan, and HA. Graphene was added to enhance the mechanical properties of the scaffolds. The rheological properties were used to identify the optimal bio-ink. The rheological properties were assessed to identify which bio-ink would be appropriate for 3D bioprinting. The results showed that the sulfate chondroitin gelatin 1:8:0.029 bio-ink was the most suitable for printing. A sulfate chondroitin, gelatin, HA, and graphene ratio of 1:8:0.02:0.06 was found to have even better water absorption, porosity, compression modulus, and cytocompatibility. Scaffolds with a graphene content of 0.06% were the most conducive to cell growth. The survival number and proliferation of cells was higher when compared to other scaffolds.

There are other technologies, such as light-activated liposomes, that permit site- and time-specific drug delivery to ocular and systemic targets. Kari et al. [113] developed a light activation technology based on indocyanine green with a hyaluronic acid coating. They synthetized HA–lipid conjugates. The light activation process improved its stability in plasma when compared to polyethylene glycol coated liposomes. The coating bound more proteins in vitreous samples. It also enriched proteins related to collagen interactions. They concluded that HA-coated liposomes could be used as a functional alternative for intravenous and ocular drug delivery.

Parolin et al. [114] have worked on the potential use of HA as an anticandidal agent against vulvovaginal candidiasis in reproductive-aged women. HMw-HA has been evaluated for its antiviral and virucidal activity [115]. The effect of HA on bacterial and fungal species was reported for Ardizzoni et al. [116]. They concluded that HA staphylococci, enterococci, *Streptococcus mutans*, two *Escherichia coli* strains, *Pseudomonas aeruginosa, Candida glabrata*, and *C. parapsilosis* experienced HA dose-dependent growth inhibition. However, *E. coli* ATCC 13768 showed no effects on the strain.

Another example is the use of HA hydrogels as an alternative treatment of symptomatic knee osteoarthritis [117]. A significant improvement in the index pain was observed after six months of HA hydrogels injections.

Carvalho et al. [118] developed patches through diffusion of aqueous solutions of HA. Samples with different concentrations of diclofenac were treated into a wet bacterial nanocellulose three-dimensional porous network. The resulting patches presented thermal stability up to 200 °C as well as being non-cytotoxic to human keratinocytes. The results suggested that oral mucosal patches could be used to treat aphthous stomatitis.

Catenacci et al. [119] prepared clotrimazole-loaded ionic polymeric micelles based on HA. The HA derivatives were prepared by the interaction of HA carboxylic groups and the amine groups of dodecyl amine and hexadecyl amine. The authors concluded that ionic interaction of the mentioned micelle ingredients turned into an amphiphilic polymer with the capacity to self-arrange in aqueous environment.

Yu et al. [120] proposed the design of a microneedle system with alginate-HA that allows for a more convenient and efficient transdermal delivery of insulin. The microneedles showed the necessary strength to penetrate the skin and an excellent degradability for the release of insulin.

Peramo et al. [121] analyzed an in vitro culture system of organotypic human skin explants in the presence or absence of external fixator pins. Their objective was to study the effect of the mixture (hyaluronic acid and dermatan sulfate) on tissues when delivered at the skin–pin interface. After two weeks, the skin specimens interfaced with fixator pins showed an increment of keratinocyte apoptosis and proliferation. This mixture could help in the preservation of the epidermal basal membrane.

Conjugates of antibiotics with natural polymers are now widely used to improve drug efficacy and safety. The application of polymeric drug delivery systems is turned in an advantage in order to minimize the required dosage and frequency of drug administration. Dubashynskaya et al. [122] developed conjugates of colistin and HA using carbodiimide chemistry. HA was dissolved in 10 mL distilled water, carbodiimide hydrochloride, and N-hydroxysuccinimide. The reaction mixture was stirred for 0.5 h at 40 °C. Cytotoxicity tests were carried out in human kidney embryonic cell line (HEK 293) and human glioblastoma cell line (T98G). They tested antimicrobial activity using the microtiter broth dilution with *P. aeruginosa* ATCC 27853. Conjugate stock solutions were prepared by diluting samples in Mueller–Hinton broth incubated for 24 h at 37 °C. The developed conjugates demonstrated reduced nephrotoxicity (HEK 293 cell line) when compared to pure CT (20–50% lower).

Dubashynskaya et al. [123] also developed a convenient synthetic method to modify colistin or vitamin B12 and HA-based conjugates with a specific targeting ligand in order to improve intestinal permeability. The conjugates obtained were evaluated using pH stability studies and in vitro colistin- release profile. The authors demonstrated the stability of the obtained conjugates under gastrointestinal conditions at pH 1, 6.8, and 7.4. The modified conjugates retained their antimicrobial activity at the level of pure colistin (minimum inhibitory concentration was 2 µg/mL) as well as the absence of *P. aeruginosa* growth. The nephrotoxicity of colistin was reduced by 30–40% when using the HEK 293 cell line. The modification of B12 improved the intestinal permeability of colistin and it had an in vivo intestinal absorption increment of 50–100%.

**Table 5:** Applications of Hyaluronic acid (HA) employed in biomaterials, pharmaceutical and delivery systems.

Product	Source	Functionality	Reference
Cryogel Scaffolds	HA Commercial	HA-based in injectable cryogel scaffolds to promote regeneration of cartilage tissue for without surgery invasive defect repair.	[103]
HA/Cs Multilayered Coatings	HA from *Streptococcus equi* sp.	Promote cell adhesion into the films to induce tumor cell capture.	[104]
Composite coating	HA Commercial	Excellent cytocompatibility.	[105]
Nanoparticles	HA Commercial	As a potential therapeutic agent for OA treatment.	[108]
Nanoparticles	HA from *Streptococcus equi*	Effects after gamma irradiation nanoparticles of HA (HA-NPs) that could diminish detrimental radiation-induced processes in lung tissue.	[109]
Hydrogel	HA Commercial	Mixing LMw and HMw which had stronger in vitro antidegradation ability as suggesting potential in regenerative medicine and tissue engineering.	[110]
Dressing with Double-Crosslinked HA-Based Hydrogels	HA Mw = 3 × 10^5^ Da	Novel double-crosslinked hydrogel this will be further explored for its application in the treatment of the diabetic foot ulcers.	[111]
3D Bio-Printing (Hybrid scaffold)	Cs, Gel, and HA	Bio-scaffolds were prepared using 3D printing technology. To support the proliferation and differentiation of mesenchymal stem cells.	[112]
Light-activated liposomes	HA 8–15 kDa	Coated liposomes for drug release, stability, protein corona formation, and mobility in the vitreous humor as alternative for intravenous and ocular drug delivery.	[113,124]
Activity HA with *Lactobacillus crispatus Lyophilised*	HA Commercial Mw 1800–2300 kDa	HA and cell free culture supernatants from *L. crispatus* BC5 to design a new therapeutic strategy to counteract vulvovaginal candidiasis.	[114]
Hydrogel (Hymovis^®^) in the treatment of symptomatic knee OA	HA (Hymovis^®^, Fidia Farmaceutici S.p.A, Abano Terme, Italy)	Novel HA-based hydrogel (Hymovis^®^) in individuals suffering from knee OA to reduce pain and improve joint function.	[117]
Nanocellulose-Based Patches	HA Mw 403.31 kDa > 95%	With Diclofenac towards aphthous stomatitis treatment with a point to a diffusion and swelling-controlled drug-release mechanism.	[118]
Ionic Polymeric Micelles	LMw 50 kDa	Micelles loaded with a poorly soluble hydrophobic antifungal drug, clotrimazole, envisaging cutaneous or vaginal application.	[119]
Microneedles	HA Commercial	Transdermal delivery of insulin, the relative pharmacologic availability and relative bioavailability of insulin from microneedle in mice.	[120]
System of organotypic human skin explants	HA Commercial	Effect on tissues of a mixture of skin–pin interface was also studied. Study in vitro to analyze cellular apoptosis and proliferation.	[121]
Hydrogel	HA Commercial	Physical mixture of Poloxamer 407, chitosan and HA for the treatment of skin and mucosal wounds with antimicrobial and biological effects proposed as a suitable vehicle against infections on skin and mucosa.	[125]
Coated	HA Commercial	Coated patch was anti-thrombotic decreased neointimal thickness both in patch venoplasty and angioplasty in a rat model.	[126]
Scaffold	HA Commercial	Powerful materials platform to better mimic the biophysical and biochemical microenvironments in ECM and elucidate the roles of mechanical cues on cell biology in 3D cell culture and direct the function and fate of stem cells.	[127]
Hydrogels	Highly purified HA made by fermentation	Adipose tissue derived mesenchymal stem cells as potential aid in articular cartilage repair for OA therapy and it could be material for cartilage regeneration.	[124]
Microneedle patch	HA Commercial	Microneedles of HA with hemagglutinins of influenza for vaccination, inducing an immune response.	[128]

Chitosan (Cs), Hyaluronic acid (HA), gelatin (Gel), Native extracellular matrix (ECM), nanoparticles of HA (HA-NPs). Osteoarthritis (OA), Molecular weight (Mw), Low Molecular weight (LMw), Hight Molecular weight (HMw).

## 4. Structure and Penetration Routes of the Skin

**Figure 2:**
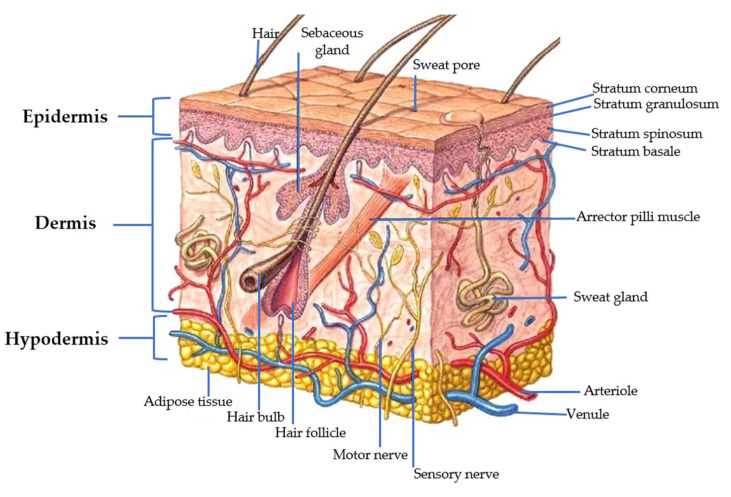
Cross-sectional scheme of the skin. The main components: epidermis, dermis, and hypodermis. Adapted from [136].

Skin and epithelial tissues have several functions (photo-protection, thermoregulation, hormonal synthesis), including a protective function as a barrier for chemical, physical, or microbial agents [129,130]. The skin has a surface area of 1.5–2 m^2^ and accounts for about 15% of the total body weight of an adult. Figure 2 shows the composition of the skin conformed for three layers: epidermis, dermis, and hypodermis. In addition, the skin contains subcutaneous fat, hair follicles, and sweat and sebaceous glands [129].

The first element is the epidermis. It is composed of the stratum corneum (SC), containing 15% water, 15% lipids, and 70% proteins [131]. The structure of the SC resembles a “brick and mortar wall”; the corneocytes are the “bricks” and are interconnected by the “mortar” of a lipid matrix. It is also composed by ceramides, cholesterol, and fatty acids [132,133]. SC thickness is approximately of 0.12 mm. The epidermis is composed of five layers: the germinative layer, the stratum spinosum, the granular layer, the lucidum layer, and the SC [134]. The basement membrane, also called the “dermal-epidermal junction”, acts as the barrier between the epidermis and the dermis [132].

The dermis possesses a thickness of around 3.5 mm. This layer provides nourishment and mechanical support to the skin [135]. In the native extracellular matrix (ECM), there are fibroblast cells and related secretion products (collagen, elastin, proteoglycans, GAG, and glycoproteins). It is formed of connective tissue, collagen fibers, and elastin [134,135].

The hypodermis (subcutaneous tissue) is the innermost layer of the skin. Its principal function is the transport of nutrients [134].

The thickness of the skin might be different depending on gender, race, age, and anatomical region. Human skin is a route for drug therapy because it avoids the hepatic metabolism route [137].

There are several types of dermal and transdermal drug delivery systems used to improve the penetration of HA in combination with other drugs. They include: microemulsions, nano emulsions, liposomes, lipid nanoparticles, lipid liquid crystals, nanocrystals, polymer nanocarriers, and inorganic nanocarriers. The use of microneedles is important in the replacement of conventional injections systems in order to administer several drugs (anti-cancer drugs, oligonucleotides, vaccines, proteins, DNA, and even nanoparticles) throughout the skin [128,138]. Microneedles have several applications in the pharmacy, medicine, and cosmetology fields. The use of these needles in medicine has grown for the administration of drugs for treatment of glaucoma. Another application has been applied to the monitoring of various biomarkers [134].

**Figure 3:**
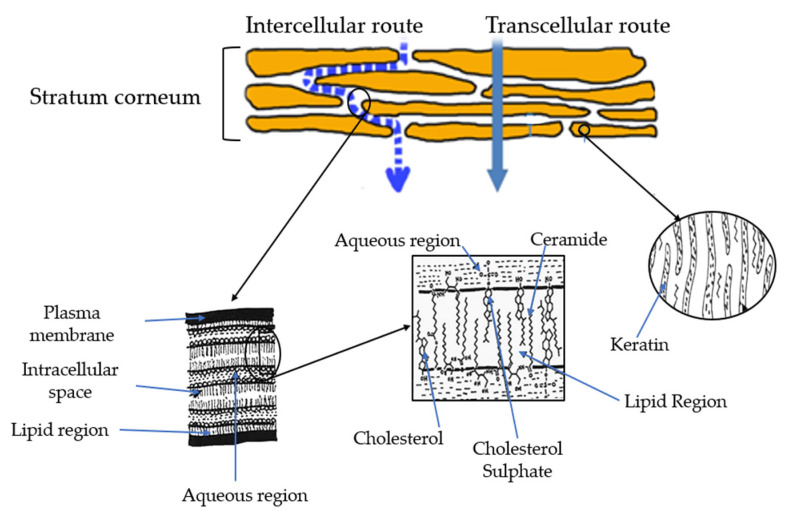
Layers of the skin and penetration routes. Adapted from [66,143,144].

Figure 3 shows the transdermal penetration routes through the SC. If transdermal penetration occurs between cells, it is called intercellular penetration. However, the transcellular route is observed through the cells. The contribution of these pathways depends mainly on the solubility, partition coefficients, and diffusivity of the drug within proteins or lipids. The rate and extent of transport in skin diffusion follows Fick’s law [139,140]. There are three different routes for penetration of transdermal drugs:

*Transappendageal route* (transport via pores): This route includes the transport of molecules through sweat glands and across hair follicles. This pathway is limited and shows smaller absorption area (~0.1%) when compared to transepidermal route [134,141].

*Intercellular route* (principal route of entry for lipophilic drugs): This is the most important route of entry of lipophilic drugs. The dense packing of proteins within the corneocytes promotes the entrance of lipophilic materials [134].

*Intracellular route* (drug mainly driven by its partition coefficient): In this route, the therapeutics move freely within the intercellular space and diffusion rates are governed largely by the lipophilicity of the drug. It means that lipophilic drugs preferably cross the SC via the intercellular domain and hydrophilic drugs can diffuse via the intracellular route [134,142].

The above-mentioned routes are important because there are several transdermal drug delivery systems. They have some advantages based on their effective systemic delivery bypassing digestive systems, patient compliance without painful injections, and easy control [145].

The main routes for nanocarriers can be identified within the routes mentioned, and are oriented to percutaneous absorption of efficacy components. Nanocarriers can penetrate skin appendages through the hair follicles and sebaceous glands in order to deliver components to surrounding tissues. The interplays between nanocarriers and the skin SC can enhance the permeability of desired components [138].

## 5. Future Trends

In recent years, HA has been used in several applications for a wide range of treatments, including medical (arthrology, cancer therapy, pneumology, odontology, ophthalmology, otolaryngology, rhinology, soft tissue regeneration, urology, wound treatment, etc.), biomaterials (scaffolds, nanoparticles, gels), pharmaceutical (e.g., transdermic systems, nanocarriers), nutritional (oral supplements, nutri-cosmeceuticals), or cosmetic field. The technique that has revolutionized prospects for HA applications is electrospinning. This method is based on the static charges produced during the stretching of the polymer fiber. Another perspective that has been gaining ground in recent years is the combination techniques of three-dimensional printing and electrospinning. For future advancements, there is a need for a better comprehension of the potential adverse effects of HA, the mechanisms of HA biological activity, and the design of intelligent drug carriers for effective diagnosis and treatment against cancer.

## 6. Conclusions

Hyaluronic acid is a glycosaminoglycan primarily found in the extracellular matrix of soft connective tissues. It is a highly hydrated component of connective, epithelial, and neural tissues. It can be extracted from different sources (animal terrestrial, animal marine, microorganisms, etc.). The most common sources are rooster comb, eggshell membrane, and tuna eyeballs. In this review, we reported on the methods of HA extraction. They included chemical, enzymatic, or combined extraction. However, significant differences depend on factors such as cost, environmental impact, yield, and level purity. Based on the exhaustive review of updated literature, there is a wide range of applications for HA due to its viscoelastic, biocompatible, antifungal, antiviral, wound healing, and tissue regeneration properties. HA has been applied in areas such as biomaterials and medical research to build scaffolds, hydrogels, 3D bio-printing hybrid scaffolds, and cryogels, the latter including nanoparticles for different purposes such as bone tissue, space-filling, nerve and brain tissue repair, or muscle regeneration.

HA has been used as an additive in the food industry (sausages, yoghurt, milk, etc.) and for oral supplementation (oral preparation, capsules, dry powder). These have become popular among consumers as nutricosmetic or cosmeceutical.

HA has been employed widely in the cosmetic industry to treat skin problems. For cosmetic formulations, HA is used for the preparation of patches or microneedles with the advantage of painless penetration of the active agents into the skin. The benefits and applications of HA depend on its molecular weight and the route of penetration into the structure of the skin. The use of nanocarriers to promote its percutaneous absorption.

## Figures and Tables

**Table 2:** Hyaluronic acid (HA) in the Food Industry.

Product	Source	Functionality	Reference
Smoked homogenized sausages with HA	Food-grade HA (94, 27%)	Effects of HA as additive and the effect properties of processed meat products.	[63]
Yoghurt supplemented with HA	HA (65%) rooster comb (Mobilee^TM^, Beriberi S.A., Palafolls, Spain)	Efficacy of the oral administration in healthy individuals with mild joint discomfort.	[64]
Milk	Commercial HA	Effect of polymer at several concentrations on various physicochemical properties of milk.	[65]
